# Assessment of Sex Disparities in Nonacceptance of Statin Therapy and Low-Density Lipoprotein Cholesterol Levels Among Patients at High Cardiovascular Risk

**DOI:** 10.1001/jamanetworkopen.2023.1047

**Published:** 2023-02-28

**Authors:** C. Justin Brown, Lee-Shing Chang, Naoshi Hosomura, Shervin Malmasi, Fritha Morrison, Maria Shubina, Zhou Lan, Alexander Turchin

**Affiliations:** 1 Division of Endocrinology, Brigham and Women’s Hospital, Boston, Massachusetts; 2 Pharmacy Department, Tufts Medical Center, Boston, Massachusetts; 3 Harvard Medical School, Boston, Massachusetts; 4 Amazon.com Inc, Seattle, Washington; 5 Center for Clinical Investigation, Brigham and Women’s Hospital, Boston, Massachusetts

## Abstract

**Importance:**

Many patients at high cardiovascular risk—women more commonly than men—are not receiving statins. Anecdotally, it is common for patients to not accept statin therapy recommendations by their clinicians. However, population-based data on nonacceptance of statin therapy by patients are lacking.

**Objectives:**

To evaluate sex disparities in nonacceptance of statin therapy and assess their association with low-density lipoprotein (LDL) cholesterol control.

**Design, Setting, and Participants:**

A retrospective cohort study was conducted from January 1, 2019, to December 31, 2022, of statin-naive patients with atherosclerotic cardiovascular disease, diabetes, or LDL cholesterol levels of 190 mg/dL (to convert to millimoles per liter, multiply by 0.0259) or more who were treated at Mass General Brigham between January 1, 2000, and December 31, 2018.

**Exposure:**

Recommendation of statin therapy by the patient’s clinician, ascertained from the combination of electronic health record prescription data and natural language processing of electronic clinician notes.

**Main Outcomes and Measures:**

Time to achieve an LDL cholesterol level of less than 100 mg/dL.

**Results:**

Of 24 212 study patients (mean [SD] age, 58.8 [13.0] years; 12 294 women [50.8%]), 5308 (21.9%) did not accept the initial recommendation of statin therapy. Nonacceptance of statin therapy was more common among women than men (24.1% [2957 of 12 294] vs 19.7% [2351 of 11 918]; *P* < .001) and was similarly higher in every subgroup in the analysis stratified by comorbidities. In multivariable analysis, female sex was associated with lower odds of statin therapy acceptance (0.82 [95% CI, 0.78-0.88]). Patients who did vs did not accept a statin therapy recommendation achieved an LDL cholesterol level of less than 100 mg/dL over a median of 1.5 years (IQR, 0.4-5.5 years) vs 4.4 years (IQR, 1.3-11.1 years) (*P* < .001). In a multivariable analysis adjusted for demographic characteristics and comorbidities, nonacceptance of statin therapy was associated with a longer time to achieve an LDL cholesterol level of less than 100 mg/dL (hazard ratio, 0.57 [95% CI, 0.55-0.60]).

**Conclusions and Relevance:**

This cohort study suggests that nonacceptance of a statin therapy recommendation was common among patients at high cardiovascular risk and was particularly common among women. It was associated with significantly higher LDL cholesterol levels, potentially increasing the risk for cardiovascular events. Further research is needed to understand the reasons for nonacceptance of statin therapy by patients and to develop methods to ensure that all patients receive optimal therapy in accordance with their preferences and priorities.

## Key Points

**Question:**

How are sex disparities in nonacceptance of statin therapy associated with control of low-density lipoprotein (LDL) cholesterol levels?

**Findings:**

In this cohort study of 24 212 adults at high cardiovascular risk, patients who accepted a statin therapy recommendation by their clinicians achieved an LDL cholesterol level of less than 100 mg/dL in a median time of 1.5 years vs 4.4 years for patients who did not accept statin therapy. Women were significantly less likely than men to accept statin therapy recommendations and achieve an LDL cholesterol level of less than 100 mg/dL.

**Meaning:**

This study suggests that patients who do not accept statin therapy have significantly higher LDL cholesterol levels; sex disparities in statin acceptance could be associated with cardiovascular risk for women.

## Teaser

This cohort study assesses sex disparities in nonacceptance of statin therapy and assess their association with low-density lipoprotein (LDL) cholesterol control.

## Introduction

The benefits of statin therapy for patients at high cardiovascular risk are well established.^1,2,3^ However, many of these individuals are not being treated with statins.^4,5,6^ This lack of statin therapy is particularly pronounced among women.^7,8,9,10,11^

The reasons for the lack of statin therapy among patients at high cardiovascular risk and for the persistent sex disparity are not fully understood and are likely multifactorial.^12,13,14,15^ One possible reason for the lack of recommended therapy that has recently come to light is nonacceptance of clinicians’ treatment recommendations by patients.^16,17^ Anecdotal evidence and patient surveys suggest that this phenomenon is also associated with the lack of indicated statin therapy,^15^ but there is a dearth of population-based studies on the subject. The extent to which nonacceptance of statins by patients is associated with the sex disparities in statin therapy is unknown.

A major reason for the paucity of research in this area is that information on nonacceptance of statin therapy recommendations by patients is not easily available. This information is typically not reflected in administrative data or structured data in the electronic health record (EHR). Instead, nonacceptance of statin therapy by patients is recorded primarily in narrative notes, requiring labor-intensive medical record reviews to study them. Over the last decade, however, technology for computational analysis of narrative electronic documents—natural language processing (NLP)—has become available, providing a powerful tool to investigate patient care processes that are documented only in narrative documents.^18,19,20^ This technology was used in the present study to test the hypothesis that nonacceptance by patients of a statin therapy recommendation is significantly associated with a lack of statin treatment among patients at high cardiovascular risk and specifically associated with sex disparities in statin therapy.

## Methods

This study was approved by the Mass General Brigham institutional review board, and the requirement for informed consent was waived because of the low risk of adverse effects to study participants. We followed the Strengthening the Reporting of Observational Studies in Epidemiology (STROBE) reporting guideline.

### Study Design

This retrospective cohort study was conducted from January 1, 2019, to December 31, 2022, among patients at high cardiovascular risk with elevated low-density lipoprotein (LDL) cholesterol levels. The study investigated the association between a patient’s decision to accept a statin therapy recommendation and the time to achieve an LDL cholesterol level of less than 100 mg/dL (to convert to millimoles per liter, multiply by 0.0259) as well as patient characteristics associated with acceptance of statin therapy.

### Study Cohort

Study patients included adults at high cardiovascular risk treated in practices affiliated with Mass General Brigham (a large integrated health care delivery network in Massachusetts founded by Brigham and Women’s Hospital and Massachusetts General Hospital) between January 1, 2000, and December 31, 2018. Patients were included if they fulfilled all of the following inclusion criteria: (1) older than 18 years; (2) diagnosis of atherosclerotic cardiovascular disease (ASCVD), diabetes, or an LDL cholesterol level of 190 mg/dL or more; (3) at least 2 primary care encounters prior to study entry; (4) no record of statin therapy prior to study entry; (5) an LDL cholesterol level of 100 mg/dL or more prior to study entry; and (6) at least 1 LDL cholesterol measurement obtained 12 months or more after study entry. Patients were excluded from analysis if they had missing demographic information or had a record of an adverse reaction to a statin prior to the first record of statin therapy.

### Exposures

A patient was entered into the study on the first date that statin therapy was recommended by a health care professional. This was defined as the earliest of (1) the first record of statin therapy (indicating initial acceptance of statin therapy by the patient) or (2) the first documented nonacceptance of statin therapy by the patient. Patients exited the study on the first of the following: (1) the date of death; (2) loss to follow-up as indicated by the absence of documentation from primary care, cardiology, or endocrinology clinicians for greater than 12 months; or (3) December 31, 2018.

### Outcome Measures

Acceptance or nonacceptance by the patient of a statin therapy recommendation by the health care professional served as the primary independent variable. Acceptance of statin therapy was indicated by a statin medication record in structured EHR data. Nonacceptance of statin therapy by the patient was ascertained from clinician documentation in narrative EHR notes using NLP. Time to LDL cholesterol control—time from study entry to the first measurement of an LDL cholesterol level of less than 100 mg/dL—served as the primary outcome. Achievement of an LDL cholesterol level of less than 100 mg/dL within 12 months of study entry was analyzed as a secondary outcome.

### Data Acquisition

Information on baseline patient characteristics and study outcomes was obtained from the EHR at Mass General Brigham. Information on participant race and ethnicity was based on self-report and was also obtained from the EHR at Mass General Brigham. The following race and ethnicity categories were obtained: Asian, Black, Hispanic, White, and other. The “other” category included American Indian or Alaska Native, Native Hawaiian or Other Pacific Islander, and unknown. Natural language processing tools to identify documented nonacceptance of statin therapy by patients were developed using the publicly available Canary platform, version 2.01.^21,22^ Canary allows users to create customized NLP tools by first defining groups of related words (word classes) that represent subconcepts of interest (eg, statins) and then defining how these word classes can come together (eg, nonacceptance of statin therapy) to describe the concept being sought. They were validated against manual annotations of 3999 randomly selected clinician notes by 2 independent reviewers whose ratings were subsequently reconciled. Additional details of development and validation of the NLP tool can be found in eFigure 2 and eAppendices 1-3 in Supplement 1. To identify patients who did not accept statin therapy, all EHR notes from clinicians in all specialties written between the date of the first eligible diagnosis and the earliest date of the first record of statin therapy, the first record of statin intolerance, or the date of study exit were analyzed.

### Statistical Analysis

An individual patient served as the unit of analysis. Univariate analyses were conducted using the *t* test for continuous variables and the χ^2^ test for categorical variables. The log-rank test was used to compare time to LDL cholesterol control between patients who initially accepted statin therapy vs patients who did not accept statin therapy.

A marginal Cox proportional hazards regression model was used to estimate the association between statin therapy acceptance vs nonacceptance and time to LDL cholesterol control while accounting for clustering within individual clinicians and adjusting for demographic confounders (age, sex, race and ethnicity, primary language, marital status, health insurance, and median income by zip code), the Charlson Comorbidity Index, indication for statin therapy (ASCVD, diabetes, or LDL cholesterol ≥190 mg/dL), family history of ASCVD or diabetes, ezetimibe use, year of the statin therapy recommendation, and the baseline LDL cholesterol level. We also conducted a sensitivity analysis (results in eAppendix 4 and eFigure 3 in Supplement 1) of how possible errors of the NLP algorithm could affect the results of this analysis.

To study the factors associated with statin therapy acceptance, a multivariable logistic regression model was constructed to estimate the association between statin therapy acceptance and patient characteristics while accounting for clustering within individual clinicians. Interpretation of *P* values was adjusted for multiple hypothesis testing using the Simes-Hochberg method.^23,24^ Analyses were performed using SAS, version 9.4 (SAS Institute Inc) and R, version 4.2.0 (R Group for Statistical Computing) for Monte Carlo simulations. All *P* values were from 2-sided tests and results were deemed statistically significant at *P* < .05.

## Results

### Natural Language Processing

We first sought to construct and evaluate the effectiveness of the NLP algorithm to identify patients not accepting of statin therapy. In the course of the evaluation of the NLP algorithm, of the 3999 notes in the validation set, 40 contained documentation of a patient not accepting a statin. The Cohen κ coefficient for interrater agreement between the 2 annotators was 0.872 (95% CI, 0.800-0.945). The NLP tool used in the analysis achieved a sensitivity of 87.5% (95% CI, 73.2%-95.8%), a specificity of 99.8% (95% CI, 99.5%-99.9%), and a positive predictive value of 77.8% (95% CI, 65.1%-86.8%).

### Study Cohort

A total of 25 197 statin-naive adults at high cardiovascular risk followed up in primary care settings, with both elevated baseline LDL cholesterol and follow-up LDL cholesterol measurements recorded, were identified. After excluding patients who (1) had a record of an adverse effect to a statin prior to the first record of statin therapy or (2) were missing data on age, sex, or median income by zip code, 24 212 patients (mean [SD] age, 58.8 [13.0] years; 12 294 women [50.8%]) were included in the study (Table 1; eFigure 1 in Supplement 1). A total of 4 575 430 EHR notes were analyzed with NLP to identify instances of statin nonacceptance by patients and their subsequent dates of study entry.

**Table 1.** Baseline Characteristics of Study Patients

Characteristic	Patients, No. (%)		*P* value
	Female (n = 12 294)	Male (n = 11 918)	
Age, mean (SD), y	60.7 (12.9)	56.8 (13.0)	<.001
Study entry year, mean (SD)^a^	9.4 (4.8)	9.7 (4.7)	<.001
Baseline LDL cholesterol, mean (SD), mg/dL	149.0 (37.9)	143.3 (35.5)	<.001
Income (in the $10 000s), mean (SD)^b^	6.9 (2.5)	7.2 (2.7)	<.001
Charlson Comorbidity Index, mean (SD)	5.2 (3.8)	4.6 (4.0)	<.001
No. of drug allergies, mean (SD)	2.8 (3.4)	1.3 (1.9)	<.001
Days of follow-up, mean (SD)	2930.3 (1668.7)	2793.6 (1632.5)	<.001
English speaking	10 321 (84.0)	10 480 (87.9)	<.001
Married	5269 (42.9)	7435 (62.4)	<.001
Government insurance	5557 (45.2)	4392 (36.9)	<.001
Smoker	4195 (34.1)	4523 (38.0)	<.001
Ezetimibe use	267 (2.2)	279 (2.3)	.38
CAD	3989 (32.5)	4770 (40.0)	<.001
CVA	1915 (15.6)	1667 (14.0)	<.001
PVD	1796 (14.6)	1590 (13.3)	.004
Diabetes	6206 (50.5)	5793 (48.6)	.004
LDL cholesterol ≥190 mg/dL	4216 (34.3)	3340 (28.0)	<.001
Family history of diabetes	3230 (26.3)	2637 (22.1)	<.001
Family history of CVD	2446 (19.9)	1963 (16.5)	<.001
Race and ethnicity			
Asian	539 (4.4)	499 (4.2)	<.001
Black	1163 (9.5)	905 (7.6)	
Hispanic	770 (6.3)	675 (5.7)	
White	8758 (71.2)	8907 (74.7)	
Other^c^	1064 (8.7)	932 (7.8)	

Abbreviations: CAD, coronary artery disease; CVA, cerebrovascular accident; CVD, cardiovascular disease; LDL, low-density lipoprotein; PVD, peripheral vascular disease.

SI conversion factor: To convert LDL cholesterol to millimoles per liter, multiply by 0.0259.

^a^ After study start in 2000.

^b^ Mean annual household income by zip code.

^c^ Included American Indian or Alaska Native, Native Hawaiian or Other Pacific Islander, and unknown.

**Figure 1.**
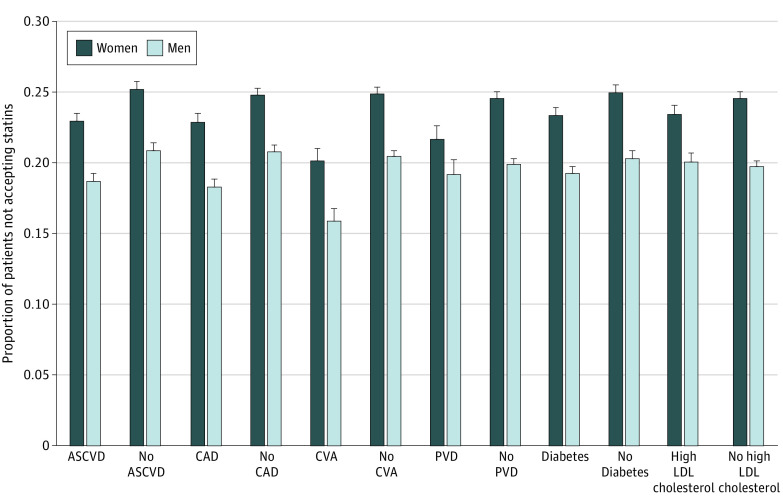
Sex Differences in Statin Acceptance Error bars indicate standard error. ASCVD indicates atherosclerotic cardiovascular disease; CAD, coronary artery disease; CVA, cerebrovascular accident; LDL, low-density lipoprotein; and PVD, peripheral vascular disease.

A total of 11 667 patients (48.2%) had ASCVD, and the rest had either diabetes or severe hypercholesterolemia; 5584 patients (23.1%) had multiple indications for statin therapy. Among study patients, 5308 (21.9%) initially did not accept statin therapy, and 1457 (6.0%) never initiated a statin during the follow-up period. Women were more likely than men to both not accept the initial statin therapy recommendation (24.1% [2957 of 12 294] vs 19.7% [2351 of 11 918]; *P* < .001) and never initiate a statin during the study (7.2% [881 of 12 294] vs 4.8% [576 of 11 918]; *P* < .001). Similar findings were observed in every subgroup in the analysis stratified by comorbidities (Figure 1). Ezetimibe use was uncommon among patients who did not accept statin therapy and was similar among women (44 of 2957 [1.5%]) and men (34 of 2351 [1.4%]) (*P* > .99). The median baseline LDL cholesterol level was 137 mg/dL. Over the mean (SD) follow-up of 7.9 (4.5) years, 18 796 patients (77.6%) reached an LDL cholesterol level of less than 100 mg/dL after a median of 1.9 years (IQR, 0.5-6.8 years).

### Nonacceptance of Statin Therapy and LDL Cholesterol Control

**Figure 2.**
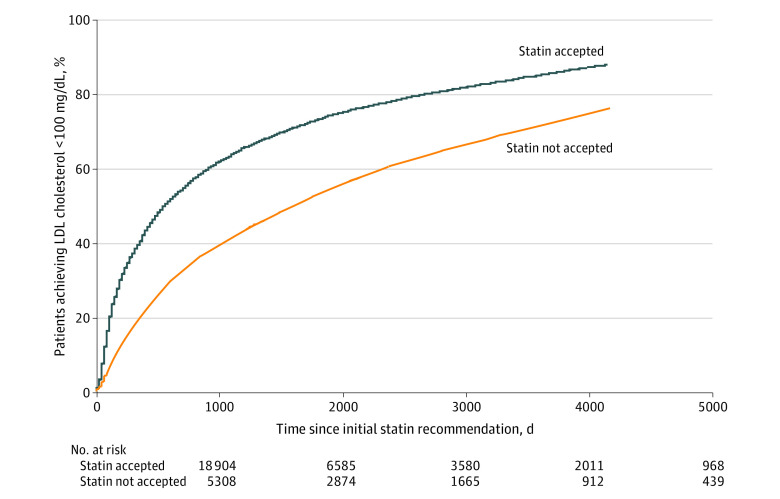
Statin Acceptance and Time to Low-Density Lipoprotein (LDL) Cholesterol Control The last 5% of the population in each group were not included in the plot due to low participant numbers and resulting wide 95% CIs. To convert LDL cholesterol to millimoles per liter, multiply by 0.0259.

Patients who accepted a statin therapy recommendation achieved an LDL cholesterol level of less than 100 mg/dL after a median of 1.5 years (IQR, 0.4-5.5 years) compared with 4.4 years (IQR, 1.3-11.1 years) (Figure 2) for patients who did not (*P* < .001). In a multivariable analysis adjusted for patients’ demographic characteristics and comorbidities, nonacceptance of statin therapy was associated with longer time to achieve LDL cholesterol control (hazard ratio [HR], 0.57 [95% CI, 0.55-0.60]; *P* < .001) (Table 2). Female sex was an independent risk factor associated with a longer time to achieve LDL cholesterol control (HR, 0.84 [95% CI, 0.81-0.87]; *P* < .001). A higher baseline LDL cholesterol level was also associated with a longer time to achieve LDL cholesterol control, while a higher Charlson Comorbidity Index, a history of diabetes or stroke, and a later study entry year were associated with a shorter time to achieve LDL cholesterol control.

**Table 2.** Patient Characteristics and Time to LDL Cholesterol Control

Parameter	Hazard ratio (95% CI)^a^	*P* value
Age	1.01 (1.01-1.01)	<.001
Study entry year	1.02 (1.02-1.03)	<.001
Baseline LDL cholesterol (per 10 mg/dL)	0.94 (0.94-0.95)	<.001
Median household income by zip code (per $10 000)	0.98 (0.97-0.99)	<.001
Charlson Comorbidity Index	1.02 (1.02-1.03)	<.001
No. of drug allergies	0.98 (0.98-0.99)	<.001
English as the primary language	0.95 (0.90-1.00)	.06
Married	1.02 (0.99-1.06)	.16
Government insurance	1.00 (0.97-1.03)	.91
Smoker	1.02 (0.98-1.05)	.31
Ezetimibe use	0.92 (0.84-1.02)	.13
CAD	0.99 (0.95-1.02)	.44
CVA	1.10 (1.06-1.15)	<.001
PVD	1.05 (1.00-1.09)	.045
Diabetes	1.21 (1.16-1.26)	<.001
History of LDL cholesterol ≥190 mg/dL	0.70 (0.67-0.74)	<.001
Family history of diabetes	1.00 (0.97-1.04)	.85
Family history of CVD	0.97 (0.93-1.01)	.09
Race and ethnicity^b^		
Asian	1.05 (0.96-1.15)	.30
Black	0.82 (0.78-0.87)	<.001
Hispanic	0.86 (0.79-0.95)	.002
Other^c^	0.94 (0.89-1.00)	.06
Female^d^	0.84 (0.81-0.87)	<.001
Statin nonacceptance	0.57 (0.55-0.60)	<.001

Abbreviations: CAD, coronary artery disease; CVA, cerebrovascular accident; CVD, cardiovascular disease; LDL, low-density lipoprotein; PVD, peripheral vascular disease.

^a^ Marginal Cox proportional hazards regression model was used to estimate the association between statin therapy acceptance vs nonacceptance and time to LDL cholesterol control while accounting for clustering within individual clinicians. All variables in the table were included in the model.

^b^ White race served as the reference (comparison) category.

^c^ Included American Indian or Alaska Native, Native Hawaiian or Other Pacific Islander, and unknown.

^d^ Male biological sex served as the reference (comparison) category.

In a secondary analysis, 42.1% (7955 of 18 904) of patients who did accept statin therapy vs 21.0% (1114 of 5308) of patients who did not accept statin therapy achieved an LDL cholesterol level of less than 100 mg/dL within 12 months (*P* < .001). In multivariable analysis, nonacceptance of statin therapy was associated with an odds ratio (OR) for achieving LDL cholesterol control within 12 months of 0.32 (95% CI, 0.30-0.35) (*P* < .001); findings of a secondary analysis using LDL decrease by 50% as the outcomes were similar (eAppendix 5 in Supplement 1). Women (independently from other characteristics) were less likely to achieve LDL cholesterol control within 12 months (OR, 0.85 [95% CI, 0.80-0.90]; *P* < .001).

### Factors Associated With Statin Therapy Acceptance

**Figure 3.**
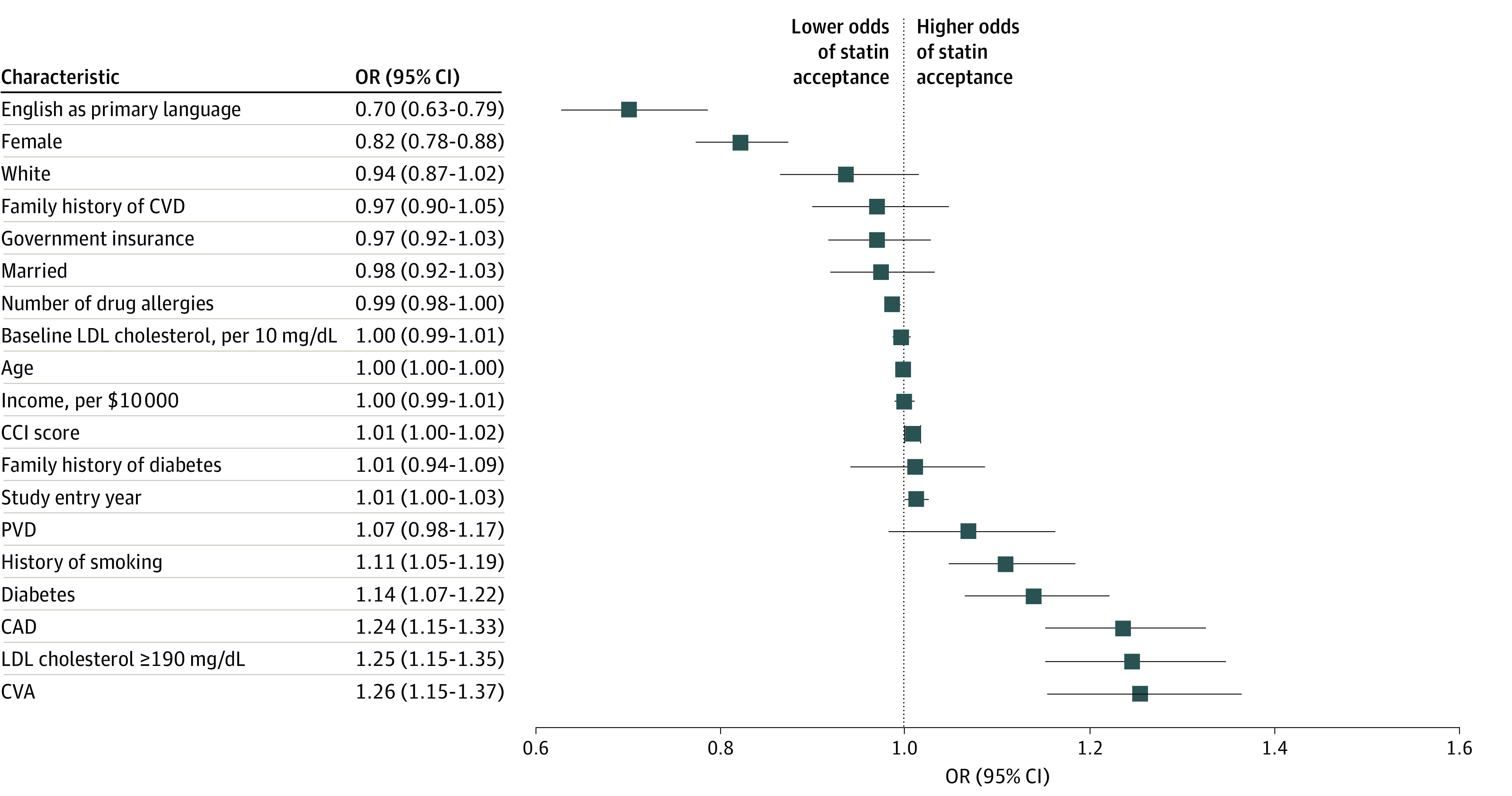
Patient Characteristics and Acceptance of Statin Therapy Recommendation A multivariable logistic regression model that included all variables in the figure was constructed to estimate the association between statin therapy acceptance and patient characteristics while accounting for clustering within individual clinicians. To convert low-density lipoprotein (LDL) cholesterol to millimoles per liter, multiply by 0.0259. CCI indicates Charlson Comorbidity Index; CAD, coronary artery disease; CVA, cerebrovascular accident; CVD, cardiovascular disease; OR, odds ratio; and PVD, peripheral vascular disease.

In multivariable analysis (Figure 3), women were less likely to agree to take a statin when first recommended by a clinician (OR, 0.82 [95% CI, 0.78-0.88]; *P* < .001). Presence of cardiovascular disease, higher baseline LDL cholesterol, and history of smoking were associated with a greater probability of statin acceptance. All of these associations were statistically significant after adjustment for multiple hypothesis testing.

## Discussion

In this large, population-based cohort study, we found that many patients with unequivocal indications for cholesterol lowering did not accept statin therapy recommended to them by their health care professionals. These patients were subsequently less likely to achieve LDL cholesterol control within 1 year and took significantly longer to reach it compared with patients who initiated statins. Women had lower rates of statin acceptance than men, potentially contributing to the known sex disparities in LDL cholesterol control. These findings shed light on an important and previously unexamined aspect of prevention of cardiovascular disease.

Nonacceptance of statin therapy recommendations is in many ways distinct from the more widely studied phenomenon of statin nonadherence. Unlike medication nonadherence, which is often due to high medication costs or adverse reactions the patient developed,^25,26,27^ nonacceptance of treatment recommendations takes place before the patient has had any direct experience with the medication; the reasons for it are therefore likely to be different. Many measures of the quality of cardiovascular care may interpret patient nonacceptance of statin therapy as “nonprescriptions” due to lack of appropriate action by the health care professional.^28,29^ This study demonstrates that patients are active agents in their care, and their preferences and priorities should be carefully taken into account when making treatment recommendations.^30,31^

Debates continue about indications for statin therapy for patients with low cardiovascular risk or for the primary prevention population.^32,33,34^ However, the present study identified high rates of nonacceptance of statin therapy among patients at high cardiovascular risk: those with existing ASCVD, diabetes, or an LDL cholesterol level of 190 mg/dL or more. These are vulnerable individuals for whom evidence-based cholesterol-lowering therapy could significantly lower the incidence of cardiovascular events and related morbidity and mortality. Therefore, the findings of this study have significant implications for public health as we continue to strive to decrease the risks of ASCVD—the number one cause of death in the US and worldwide.^35,36^

Acceptance of a statin therapy recommendation was found to be associated with achievement of LDL cholesterol control. This association is not as self-evident as it may seem because patients’ cholesterol levels may have been associated with many other factors. On the one hand, a patient’s initial acceptance of statin therapy does not necessarily guarantee continuous use of the statin, or even any use at all, because patients can often be nonadherent to statins.^37,38^ On the other hand, a patient’s initial nonacceptance does not necessarily indicate that the patient will never take a statin—nearly two-thirds of patients who initially did not accept a statin did eventually start taking statins, which may have occurred shortly after their initial nonacceptance. Finally, both groups of patients may have also elected to use other nonstatin cholesterol-lowering medications, such as ezetimibe, or implemented lifestyle changes that may lead to lower cholesterol levels. However, the association of statin nonacceptance with LDL cholesterol levels was marked despite these potential considerations.

The present study identified significant sex disparities in the acceptance of statin therapy. Women were more than 20% more likely than men not to accept their clinician’s initial statin therapy recommendation; similar findings were observed among patients with known coronary artery disease and all other comorbidity subgroups. This disparity increased as time went on; over the entire course of the study, women were 50% more likely to never initiate statins. Multiple previous studies have reported lower rates of cholesterol control among women compared with men.^39,40,41^ These differences have been explained in part by sex disparities in the rates of adherence and adverse effects to statins.^13,42,43,44^ The present study suggests that disparities in nonacceptance of a statin therapy recommendation are another important factor; further research is needed to assess why women at high cardiovascular risk are less likely to accept their clinicians’ recommendation of statin therapy.

A distinct aspect of this study was the use of novel NLP technology to identify patients who did not accept a statin therapy recommendation by their clinician. Nonacceptance of statin therapy by patients is typically documented only in narrative clinician notes because EHR systems do not usually have checkboxes, drop-down lists, or other structured data elements where it could be recorded. Data analysis for the study involved over 4 million electronic clinician notes, rendering traditional manual medical record review infeasible. On the other hand, the validated NLP tool developed for the purpose of this study processed this massive amount of data within days. This study therefore highlights the potential for the use of artificial intelligence technology in combination with vast data sets to make novel research questions accessible for investigation for the first time.

### Strengths and Limitations

The present study has several strengths. To our knowledge, this is the first population-based study that examined nonacceptance of statin therapy recommendations by patients. It included a large population of patients treated in primary care settings, similar to most patients with hypercholesterolemia in the US. The study focused on patients with unequivocal indications for statin therapy, making its findings critical for the prevention of cardiovascular events among this high-risk population. Finally, the use of artificial intelligence NLP technology allowed for a unique viewpoint into a previously minimally explored aspect of the treatment of hypercholesterolemia.

Study findings should also be interpreted in light of its limitations. Its observational nature does not allow for the identification of causal relationships. Some of the statin nonacceptance may not have been documented in clinician notes, and other instances may not have been detected by the NLP algorithm. Validation of the NLP algorithm was not stratified by biological sex, and it is possible that the accuracy of the algorithm is different for female vs male patients. Only the first statin therapy recommendation for each patient was examined; future studies should address whether similar findings hold for multiple recommendations. Specific reasons for statin nonacceptance, discussion of lifestyle changes, and association of clinician characteristics were not examined and should be investigated in future research. Although a broad selection of potential confounding variables was included in the multivariable analysis, other unknown confounders not accounted for may have influenced the association between statin acceptance and LDL cholesterol control. In addition, because our study cohort included only patients who were known to have obtained an LDL cholesterol level measurement after a statin recommendation, it may be biased by the exclusion of patients who did not return for follow-up care. Finally, the study population is composed of encounters within a single-center health care system; the patient population therefore may not be generalizable to other geographic or demographic cohorts.

## Conclusions

In this cohort study, nonacceptance of a statin therapy recommendation was common among patients at high cardiovascular risk and was associated with higher LDL cholesterol levels, potentially translating into an increased incidence of cardiovascular events. Nonacceptance of statins was particularly prevalent among women, possibly contributing to the known sex disparities in treatment of high cholesterol. Further research is needed to identify the reasons why patients do not accept statin therapy recommendations and the reasons for the higher rates of this important clinical phenomenon among women.

## Notes

Supplement 1. **eFigure 1.** Patient Flow **eAppendix 1.** Natural Language Processing Technology **eFigure 2.** Screenshot of Canary Natural Language Processing Platform **eAppendix 2.** Development of Natural Language Processing Tool for Identification of Statin Non-Acceptance by Patients **eAppendix 3.** Evaluation of Natural Language Processing Tools **eAppendix 4.** Sensitivity Analysis of the Effect of Possible Errors Potentially Introduced by the NLP Algorithm **eFigure 3.** Hazard Ratio Estimates Obtained by the NLP Accuracy Sensitivity Analysis **eAppendix 5.** Secondary Analysis of 50% LDL Decrease at 12 Months

Supplement 2.
Data Sharing Statement
