# Supplementary material for: Assessment of Sex Disparities in Nonacceptance of Statin Therapy and Low-Density Lipoprotein Cholesterol Levels Among Patients at High Cardiovascular Risk
Source: JAMA Netw Open. 2023 Feb 28;6(2):e231047. doi: 10.1001/jamanetworkopen.2023.1047 (PMC9975905; doi:10.1001/jamanetworkopen.2023.1047)
Supplement: Supplement 1. — eFigure 1. Patient Flow eAppendix 1. Natural Language Processing Technology eFigure 2. Screenshot of Canary Natural Language Processing Platform eAppendix 2. Development of Natural Language Processing Tool for Identification of Statin Non-Acceptance by Patients eAppendix 3. Evaluation of Natural Language Processing Tools eAppendix 4. Sensitivity Analysis of the Effect of Possible Errors Potentially Introduced by the NLP Algorithm eFigure 3. Hazard Ratio Estimates Obtained by the NLP Accuracy Sensitivity Analysis eAppendix 5. Secondary Analysis of 50% LDL Decrease at 12 Months [file jamanetwopen-e231047-s001.pdf]

Supplementary Online Content

Brown CJ, Chang LS, Hosomura N, et al. Assessment of sex disparities in nonacceptance of statin therapy and low-density lipoprotein cholesterol levels among patients at high cardiovascular risk. *JAMA Netw Open.* 2023;6(2):e231047. doi:10.1001/jamanetworkopen.2023.1047

eFigure 1. Patient Flow
eAppendix 1. Natural Language Processing Technology
eFigure 2. Screenshot of Canary Natural Language Processing Platform
eAppendix 2. Development of Natural Language Processing Tool for Identification of Statin Non-Acceptance by Patients
eAppendix 3. Evaluation of Natural Language Processing Tools
eAppendix 4. Sensitivity Analysis of the Effect of Possible Errors Potentially Introduced by the NLP Algorithm
eFigure 3. Hazard Ratio Estimates Obtained by the NLP Accuracy Sensitivity Analysis
eAppendix 5. Secondary Analysis of 50% LDL Decrease at 12 Months

This supplementary material has been provided by the authors to give readers additional information about their work.

© 2023 Brown CJ et al. *JAMA Network Open.*

eFigure 1. Patient Flow

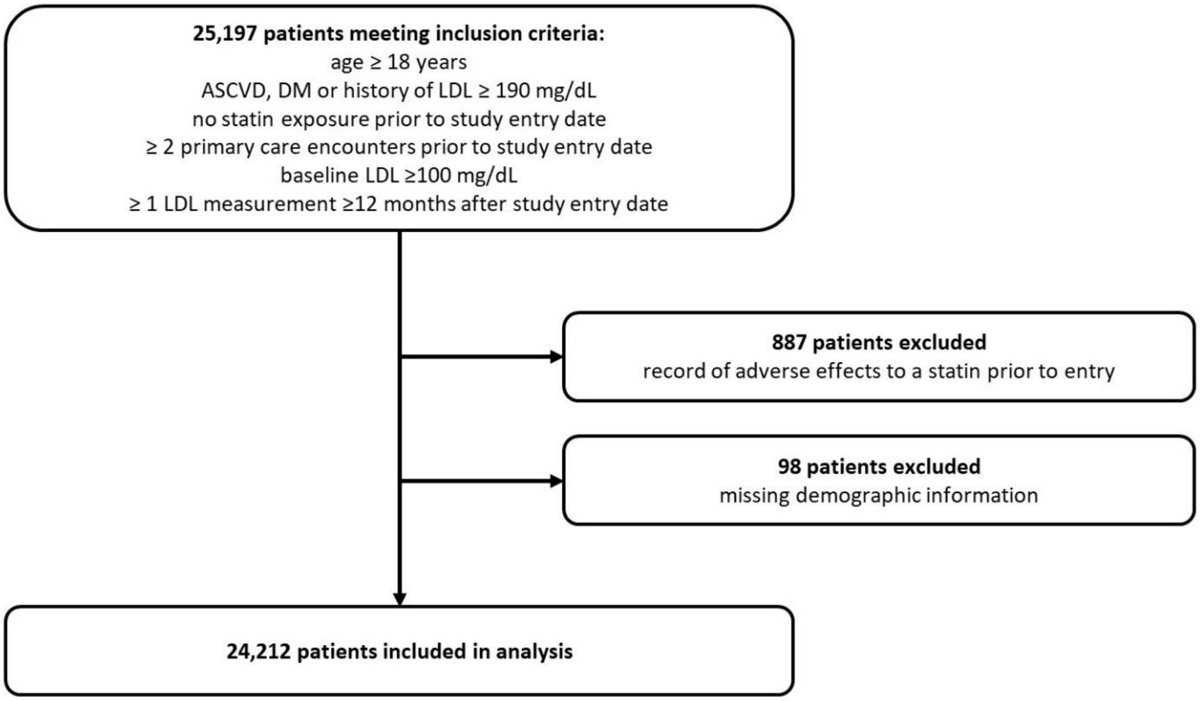

© 2023 Brown CJ et al. *JAMA Network Open.*

eAppendix 1. Natural Language Processing Technology

Natural language processing tool utilized in this study was created using the Canary software. Canary is a publicly available (http://canary.bwh.harvard.edu/) graphic user interface-based platform that aims to allow users without computer science or software engineering background create their own natural language processing tools. The main steps in building a Canary-based natural language processing tool include:

1. Preprocessing for text normalization and mapping of acronyms and synonyms; these transformations simplify the text matching process.
2. Building task-specific vocabulary through creation of user-specified semantic categories (word classes). This functionality plays an important role in supporting creation of high-fidelity language models beyond what's available through standard ontologies (though standard ontologies can also be used as Canary facilitates import of UMLS subsets). This approach allows inclusion of common misspellings or words that would only be included in this semantic category in the specific context of the concept being sought. For example, "call in" [a prescription to the pharmacy] can be considered a semantic equivalent of "prescribe" in the context of medication management, but would not be found in this category in a standard ontology, such as SNOMED.
3. Creation of grammar rules that define how these word classes can be combined to form target phrases.
4. Definition of specific conditions that must be met for information to be extracted. For example, users can specify the presence of one or more phrases, such as a medication class and adverse reaction, as an output condition.

© 2023 Brown CJ et al. *JAMA Network Open.*

eFigure 2. Screenshot of Canary Natural Language Processing Platform

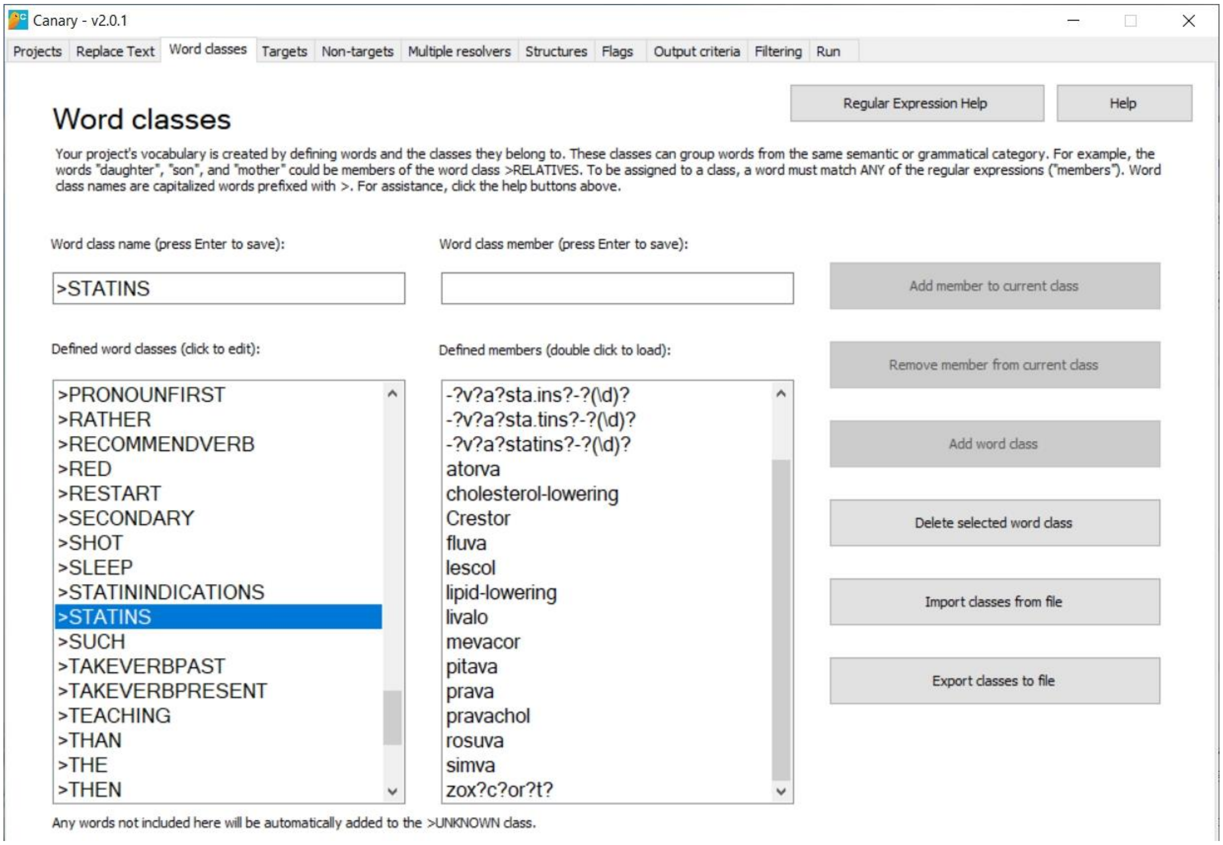

© 2023 Brown CJ et al. *JAMA Network Open.*

eAppendix 2. Development of Natural Language Processing Tool for Identification of Statin Non-Acceptance by Patients

The natural language processing tool was designed by examination of documentation of statin non-acceptance by patients identified through manual annotation of randomly selected ambulatory progress notes of patients at high cardiovascular risk who were not prescribed statins (the *training* dataset). Annotation was carried out by trained senior pharmacy students. Annotators were instructed to identify phrases that described statin-naïve patients not accepting their healthcare provider's recommendation of statin therapy. Some examples of text identified in this way include:

- *George comes in today with his wife and daughter for follow-up. They have talked and they have discussed not starting a statin for George despite a recent MI, given his age and general medical condition.*
- *He is not on statin. Again I have reviewed the risk and benefit with the family including the daughter who is a nurse and does not want to start this*.
- *Again, she declines statin therapy.*
- *I took the opportunity to again tell her how important I think taking a statin would be, but did not get anywhere.*

Annotators were instructed not to include patients who did not accept statin therapy because of their prior experience with statins (e.g. adverse reactions). A total of 20,974 notes were annotated and 263 instances of documentation of statin non-acceptance by patients were identified in the training dataset. The natural language processing tool was designed using an iterative process whereupon it was evaluated against a subset of the training dataset; false positive and false negative errors were noted and the natural language processing tool updated to correct them; and then the process was repeated on the next subset. The final version of the natural language processing tool is publicly available in the Canary NLP Tool Library at http://canary.bwh.harvard.edu/library/.

© 2023 Brown CJ et al. *JAMA Network Open.*

eAppendix 3. Evaluation of Natural Language Processing Tools

The final version of the natural language processing tool was performed against a manually annotated validation set of 3,999 randomly selected ambulatory progress notes of patients at high cardiovascular risk who were not prescribed statins (i.e. the same population as the one from which the training dataset was drawn). This *validation* dataset was selected in such a way as to not overlap with the training dataset. Each document was reviewed by two annotators and differences between their annotations were subsequently reconciled. The annotators for the validation dataset were selected from among the annotators for the (larger) training dataset and received identical instructions for the annotation. The final annotation of the validation dataset included a total of 40 instances of documentation of patients not accepting their providers' statin therapy recommendation.

© 2023 Brown CJ et al. *JAMA Network Open.*

eAppendix 4. Sensitivity Analysis of the Effect of Possible Errors Potentially Introduced by the NLP Algorithm

We conducted a sensitivity analysis to determine the effect of possible errors by the natural language processing algorithm on the results of the Cox model analysis using Monte Carlo simulations. For each of 100 simulations, we conducted two manipulations of the binary variable representing statin non-acceptance (none of the other variables in the dataset were altered) as follows: a) among the patients that were identified by the NLP algorithm to not have accepted statin therapy, we randomly selected n.P × (1-PPV) patients (where n.P is the number of patients with NLP-derived statin non-acceptance and PPV is the positive predictive value of the NLP algorithm) and changed the value of the variable to "statin therapy accepted"; and b) among the patients that were not identified by the NLP algorithm to not have accepted statin therapy, we randomly selected n.N*(1-NPV) patients (where n.N the number of patients with NLP-derived acceptance and NPV is the value of negative predictive value) and changed the value of the variable to "statin therapy not accepted". We then fit the resulting 100 semi-synthetic datasets to the same Cox regression model as in the original analysis and compared the distribution of the 100 hazard ratio estimates for the variables representing a) statin non-acceptance and b) female biological sex to the hazard ratios obtained in the original model.

© 2023 Brown CJ et al. *JAMA Network Open.*

eFigure 3. Hazard Ratio Estimates Obtained by the NLP Accuracy Sensitivity Analysis

A. Statin Therapy Non-Acceptance

B. Female biological sex

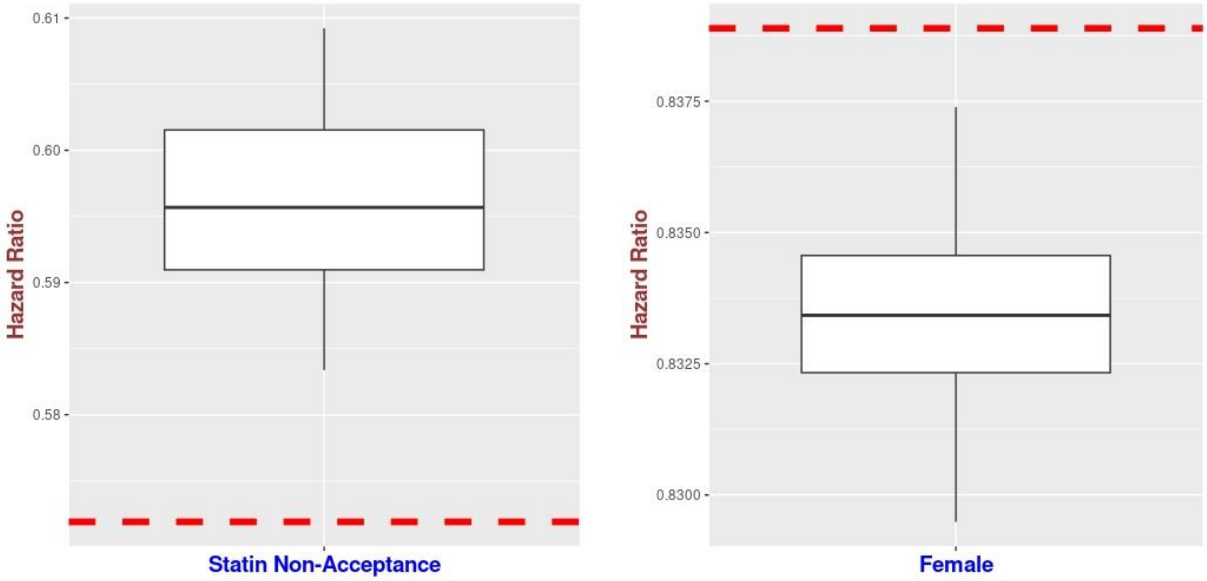

In each panel, the 100 hazard ratio estimates obtained from the 100 simulations of the original Cox analysis using the semi-synthetic dataset are visualized by a boxplot that shows their mean and the interquartile range. The red dashed line is the hazard ratio estimate based on the original data.

In this sensitivity analysis, the hazard ratio estimates obtained in the course of 100 simulations were similar to the ones in the original analysis. The mean hazard ratio for the relationship of statin therapy non-acceptance and time to LDL control was 0.596 (interquartile range 0.591 to 0.602) and the mean hazard ratio for the relationship of female sex and time to LDL control was 0.833 (interquartile range 0.832 to 0.835).

© 2023 Brown CJ et al. *JAMA Network Open.*

eAppendix5. Secondary Analysis of 50% LDL Decrease at 12 Months

We conducted a secondary analysis using achievement of LDL decrease by 50% from baseline at 12 months as the (binary) outcome. In this analysis, patients who accepted statin therapy recommendation were more likely to have their LDL decrease by 50% (2,473 of 18,904; 13.1%) compared to patients who did not (260 of 5,308; 4.9%) ($P < 0.001$). We also conducted a multivariable analysis by constructing a multivariable logistic regression model that included all independent variables included in the primary analysis and was adjusted for clustering within providers. In this multivariable analysis, non-acceptance of statin therapy by patients was associated with odds ratio of 0.344 (95% CI 0.299 to 0.396; $P <0.001$) and female sex with odds ratio of 0.784 (95% CI 0.718 to 0.857; $P < 0.001$) for achievement of LDL decrease of 50% at 12 months.

© 2023 Brown CJ et al. *JAMA Network Open.*
