# Supplementary material for: Assessment of Sex Disparities in Nonacceptance of Statin Therapy and Low-Density Lipoprotein Cholesterol Levels Among Patients at High Cardiovascular Risk
Source: JAMA Netw Open. 2023 Feb 28;6(2):e231047. doi: 10.1001/jamanetworkopen.2023.1047 (PMC9975905; doi:10.1001/jamanetworkopen.2023.1047)
Supplement: Supplement 2. — Data Sharing Statement [file jamanetwopen-e231047-s002.pdf]

Data Sharing Statement

Brown. Assessment of Sex Disparities in Nonacceptance of Statin Therapy and Low-Density Lipoprotein Cholesterol Levels Among Patients at High Cardiovascular Risk. *JAMA Netw Open*. Published February 28, 2023. doi:10.1001/jamanetworkopen.2023.1047

Data
Data available: Yes
Data types: Deidentified participant data
How to access data: De-identified participant data will be available by request to the corresponding author Dr. Alexander Turchin (aturchin@bwh.harvard.edu), subject to the institutional policies.
When available: With publication

Supporting Documents
Document types: None

Additional Information
Who can access the data: Anyone requesting the data
Types of analyses: Any purpose
Mechanisms of data availability: With a signed data access agreement as per institutional policy.
